# Patient and caregiver perspectives on transition from pediatric to adult care in inborn errors of immunity: a Polish multicenter survey

**DOI:** 10.3389/fimmu.2026.1816943

**Published:** 2026-06-26

**Authors:** Agata Będzichowska, Agata Tomaszewska, Katarzyna Napiórkowska-Baran, Aleksandra Matyja-Bednarczyk, Marcin Ziętkiewicz, Sylwia Kołtan, Karina Jahnz-Różyk, Ewa Więsik-Szewczyk

**Affiliations:** 1 Department of Internal Medicine, Pneumonology, Allergology, Clinical Immunology and Rare Diseases, Military Institute of Medicine – National Research Institute, Warsaw, Poland; 2 Department of Pediatrics, Pediatric Nephrology and Allergology, Military Institute of Medicine – National Research Institute, Warsaw, Poland; 3 Faculty of Medicine, University of Warsaw, Warsaw, Poland; 4 Department of Allergology, Clinical Immunology and Internal Diseases, Ludwik Rydygier Collegium Medicum in Bydgoszcz Nicolaus Copernicus University in Torun, Bydgoszcz, Poland; 5 Center for Transplantology and Interstitial Lung Diseases, Collegium Medicum, Jagiellonian University, Krakow, Poland; 6 Department of Rheumatology, Clinical Immunology, Geriatrics and Internal Medicine, Medical University of Gdansk, Gdansk, Poland; 7 Department of Paediatrics, Haematology, Oncology, Immunology and Transplantology, Ludwik Rydygier Collegium Medicum in Bydgoszcz Nicolaus Copernicus University in Torun, Bydgoszcz, Poland

**Keywords:** brief illness perception questionnaire, caregivers, inborn errors of immunity, primary immunodeficiency, transition care, young adults

## Abstract

**Introduction:**

Advances in the diagnosis and treatment of inborn errors of immunity (IEI) have improved survival rates, resulting in a growing population of adolescents requiring transition from pediatric to adult immunology care. However, data reflecting real-world transition practices remain limited. This multicenter survey aimed to assess current patient and caregiver experiences of the transition process in Poland.

**Materials and methods:**

The analysis included data collected from adult patients with IEI and their parents or caregivers after transition to adult care. A structured, study-specific questionnaire developed by the authors was used to assess characteristics of the transition process and satisfaction with care. Illness perception was evaluated using the validated Brief Illness Perception Questionnaire (B-IPQ).

**Results:**

A total of 50 patients with inborn errors of immunity and 18 parents or caregivers were included in the analysis. Lack of transition preparation was significantly associated with a greater emotional burden of illness, with unprepared patients reporting higher scores in the Emotional Representation domain of the B-IPQ compared with prepared patients (6.42 vs. 4.06; p = 0.019). A trend toward greater perceived personal control over illness was observed among prepared patients (p = 0.050). Prepared patients more frequently attended medical appointments without parental presence (80.6% vs. 50.0%, p = 0.033). Patients with comorbidities reported significantly greater symptom severity (p = 0.004) and greater impact of illness on daily functioning (p = 0.028) compared with patients without comorbidities. Despite this higher disease burden, patients with comorbidities reported lower rates of transition preparation (65.4% vs. 79.2%). Comparison of patient and parent perspectives demonstrated high concordance in reported transition preparation rates and ratings of adult care quality. However, patients rated the transition process more positively than their parents. The most frequently reported difficulties during transition included poor coordination between pediatric and adult centers, insufficient engagement of medical staff, and lack of psychological support.

**Conclusions:**

This first Polish study demonstrated a statistically significant association between transition preparation and lower emotional burden of illness among young adults with IEI. Patients who were not prepared for transition reported a greater negative emotional impact of their condition. These findings provide empirical support for the development of national transition guidelines for IEI in Poland, including structured transition preparation, dedicated transition coordinator roles, and the integration of psychosocial support into routine care.

## 1 Introduction

Inborn errors of immunity (IEI) comprise a heterogeneous group of approximately 500 rare genetic disorders characterized by impaired immune system function (1–3). The clinical spectrum of IEI is broad and may include recurrent infections, autoimmune diseases, allergic manifestations, autoinflammation, and an increased risk of malignancy (1, 2, 4).

Advances in diagnostic methods and treatment strategies have substantially improved survival among patients with IEI, resulting in an increasing number of individuals reaching adulthood and requiring long-term specialist care (2, 5–7). In Poland, despite the absence of a national IEI registry, it is estimated that several thousand individuals live with these conditions, many of whom are currently transitioning—or will soon transition—from pediatric to adult healthcare services (8).

Immunology care for children with IEI in Poland is concentrated in specialized pediatric centers, whereas adult care remains more fragmented and less structured. The absence of national transition guidelines for IEI, together with the lack of systematic monitoring of the transition process, contributes to considerable inter-center variability in clinical practice (6, 9, 10).

Transition is defined as a planned, gradual process that prepares a young patient to function within the adult healthcare system, including medical, psychosocial, and educational aspects (11, 12). In contrast, transfer refers specifically to the administrative event of moving from pediatric to adult care, which constitutes only one element of the transition process (6, 10). In IEI, transition is particularly important because of the chronic nature of these conditions, the need for continuous treatment, and the risk of severe complications associated with disrupted continuity of care (6, 7, 10). This process also coincides with a critical stage of psychosocial development, during which patients are expected to assume increasing responsibility for managing their own health (11, 13).

Despite its growing importance, data regarding transition experiences among patients with IEI—particularly within the Polish healthcare context—remain limited (6, 9, 10, 14). Therefore, the aim of this study was to assess the experiences of young adults with IEI and their parents regarding the transition process in Poland, and to identify key barriers and unmet needs. The findings may contribute to improving the organization of healthcare services for this population.

## 2 Materials and methods

A retrospective, questionnaire-based observational study was conducted to assess experiences related to the transition process in Poland. The study included both the perspectives of patients and their parents or caregivers. The study protocol was approved by the Bioethics Committee of the Military Institute of Medicine – National Research Institute in Warsaw (No. 36/WIM/2024).

Participants were recruited from four major academic adult immunology centers in Poland: Warsaw (Military Institute of Medicine – National Research Institute), Bydgoszcz (Ludwik Rydygier Collegium Medicum, Nicolaus Copernicus University), Cracow (Jagiellonian University Medical College), and Gdansk (Medical University of Gdansk). These tertiary referral centers were selected because of their established adult IEI programs, providing specialized care across the major geographic regions of Poland. Eligible participants were adults (≥18 years) with a confirmed diagnosis of IEI requiring immunoglobulin replacement therapy and/or immunosuppressive or biological treatment who had completed the transition to adult immunology care. Patients were recruited consecutively during scheduled outpatient visits between January and September 2025. Eligible individuals were identified and informed about the study by their treating immunologist. Written informed consent was obtained prior to questionnaire administration.

### 2.1 Study instruments

A study-specific anonymous questionnaire was developed for this study and administered in either paper or electronic format, according to participant preference. As no standardized transition assessment tool for IEI was available at the time of study design, the questionnaire was developed by the research team based on international transition care recommendations and expert consensus among the co-authors. The instrument was not formally validated and did not undergo pilot testing. It was designed to be self-explanatory, although participants could seek clarification from clinical staff if needed. The full questionnaire is provided in the **Supplementary Materials**. The questionnaire collected demographic and clinical data, information on the transition process, and assessments of satisfaction with both transition and adult care. In addition, the validated Polish version of the Brief Illness Perception Questionnaire (B-IPQ) was administered to assess cognitive and emotional perceptions of illness. The B-IPQ uses a 0–10 scale, where 0 indicates the lowest and 10 the highest intensity of the assessed domain. A parallel questionnaire for parents or caregivers included analogous questions but did not include the B-IPQ.

### 2.2 Statistical analysis

Continuous variables were presented as means ± standard deviation or medians with interquartile ranges, as appropriate. Categorical variables were expressed as counts and percentages. Prior to analysis, data were preliminarily assessed with normality plots and subsequently tested using the Kolmogorov–Smirnov test with Lilliefors correction. Given that some variables did not follow a normal distribution, non-parametric tests not requiring normality were applied. Student’s t-test was used for variables with a normal distribution. Group comparisons were performed using the Mann–Whitney U test for continuous variables and the chi-square test or Fisher’s exact test for categorical variables. B-IPQ domains were analyzed separately, without calculating a total score. Statistical significance was set at p = 0.05. All analyses were performed using SPSS Statistics for Windows, version 29.0.0.0 (IBM Corp., New York, USA).

## 3 Results

### 3.1 Characteristics of the study population

**Figure 1:**
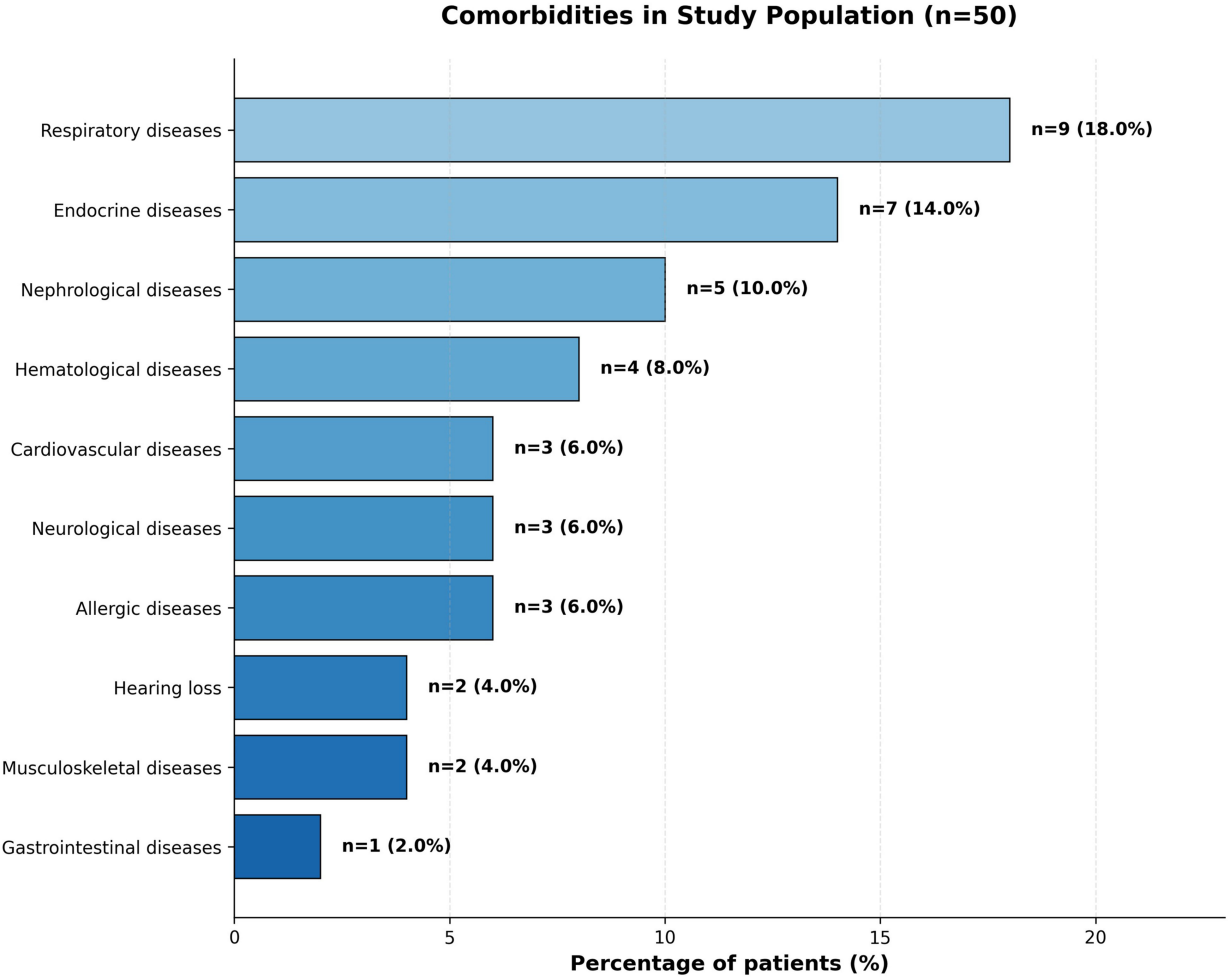
Comorbidities in the patient study group.

The study included 50 young adult patients diagnosed with inborn errors of immunity (IEI) and 18 parents or caregivers of patients who had undergone the transition process between 2015 and 2025. The mean age of patients at the time of the study was 25 years (± 6.15), and males constituted the majority of the study population (67.3%). Comorbidities were reported by 26 patients (52%), most commonly involving the respiratory and endocrine systems. Detailed demographic and clinical characteristics are presented in **Table 1**, **Figure 1**.

**Table 1:** Characteristics of study participants.

Variable	Patients (n = 50)	Parents/caregivers (n = 18)
Age, years	25 ± 6.15	56 ± 5.17
Female sex^#^, n (%)	16 (32.7)	14 (77.8)
Male sex^#^, n (%)	33 (67.3)	4 (22.2)
Rural residence, n (%)	11 (25.6)	5 (27.8)
City <50,000, n (%)	6 (14.0)	2 (11.1)
City 50–150,000, n (%)	7 (16.3)	5 (27.8)
City 100–500,000, n (%)	7 (16.3)	4 (22.2)
City >500,000, n (%)	12 (27.9)	2 (11.1)
Primary education, n (%)	7 (14.9)	0 (0.0)
Secondary education, n (%)	27 (57.4)	4 (22.2)
Higher education, n (%)	13 (27.7)	14 (77.8)
≥1 comorbidity, n (%)	26 (52.0)	–

^#^Percentages calculated excluding missing data (n = 1).

### 3.2 Course of the transition process

The median age at the start of transition was 17 years (IQR 17–17). The formal transfer always took place on the day of the patient’s 18th birthday. The interval between the end of pediatric care and the first visit to an adult immunology center was ≤3 months in 71% of patients. A delay of >3 to 6 months was reported by 12.5% of patients (n = 6), while >6 to 12 months by 6.3% (n = 3). A gap of >12 to 24 months occurred in 4.2% (n = 2), and delays exceeding 24 months were reported by 6.3% (n = 3). Overall, 10.5% of patients experienced a disruption in care lasting longer than 12 months.

The opportunity to choose an adult immunology center was reported by 22/50 (44%) patients. The most frequently reported factors influencing this decision were recommendations from the treating physician (n = 19/50, 38%) and distance from the patient’s place of residence (n = 15/50, 30%). Less commonly, patients considered the center’s reputation (n = 6/50, 12%) and access to multidisciplinary care (n = 6/50, 12%). Recommendations from other patients were reported least frequently (n = 3/50, 6%).

### 3.3 Transition preparation and patient experiences

Preparation for transition from pediatric to adult care was reported by 36 patients (72%). Among prepared patients, the most common form of preparation was a conversation with a doctor (n = 19). Conversations with a nurse and contact with the adult care center were each reported by 13 patients. Fewer patients reported a personal visit to the adult center (n = 6) or the use of educational materials (n = 6). Only one patient reported a consultation with a psychologist.

Prepared patients rated the overall transition process higher than unprepared patients (mean score 7.89 vs. 5.29). They tended to have a shorter median interval between pediatric and adult visits. Both differences showed a trend toward statistical significance (p = 0.078 and p = 0.088, respectively). No significant differences were observed in the assessment of adult care quality.

Prepared patients more often attended medical visits without a parent (80.6% vs. 50%, p = 0.033), indicating greater independence in managing their healthcare (**Table 2**).

**Table 2:** Comparison of key transition care parameters between prepared and unprepared patients (patient perspective).

Parameter	Prepared (n = 36)	Unprepared (n = 14)	p-value
Age at first adult visit, years	18.2 ± 1.23 (18)	18.9 ± 3.38 (18)	0.914
Interval pediatric–adult visit, months	3.22 ± 5.00 (2.0)	9.88 ± 11.26 (3.2)	0.088
Transition process rating (1–10)	7.89 ± 1.77 (8.0)	5.29 ± 4.01 (6.0)	0.078
Adult care rating (1–10)	8.83 ± 1.50 (9.0)	7.93 ± 2.73 (9.0)	0.445
Visit without parent, n (%)	29 (80.6)	7 (50.0)	**0.033**

Continuous variables presented as mean ± SD (median). Categorical variables as n (%). Mann–Whitney U test for continuous variables; chi-square or Fisher’s exact test for categorical variables.

Bold values indicate statistical significance (p < 0.05).

### 3.4 Illness perception (B-IPQ)

Analysis of illness perception showed that IEI were perceived as chronic conditions (median score of 10 in the Timeline domain), with a moderate impact on daily functioning and emotional well-being.

Patients who were not prepared for transition reported a significantly greater emotional burden. The mean score in the Emotional Representation domain of the B-IPQ was 6.42 among unprepared patients, compared with 4.06 among prepared patients (p = 0.019). No significant differences were observed across the remaining B-IPQ domains (**Table 3**).

**Table 3:** Comparison of illness perception (B-IPQ) scores between prepared and unprepared patients.

B-IPQ domain (0–10)	Prepared mean ± SD (median)	Unprepared mean ± SD (median)	p-value
Impact of illness	5.41 ± 2.43 (6.0)	6.92 ± 2.91 (8.0)	0.056
Timeline	9.50 ± 1.40 (10.0)	8.67 ± 2.35 (10.0)	0.154
Personal control	6.76 ± 2.69 (8.0)	5.25 ± 2.30 (5.0)	0.050
Treatment control	5.85 ± 2.71 (6.0)	6.75 ± 2.42 (6.5)	0.372
Symptom severity	4.18 ± 2.74 (4.0)	5.83 ± 3.21 (6.5)	0.104
Concern	4.26 ± 2.55 (5.0)	4.17 ± 2.29 (4.5)	0.840
Illness coherence	6.67 ± 2.23 (7.0)	5.17 ± 2.98 (5.0)	0.133
Emotional representation	4.06 ± 2.68 (4.0)	6.42 ± 2.43 (6.5)	**0.019**

Values are mean ± SD (median). Mann–Whitney U test.

Bold values indicate statistical significance (p < 0.05).

Patients with comorbidities reported significantly greater symptom severity and disease burden (p = 0.004), as well as a greater impact of the disease on daily functioning (p = 0.028). Despite this higher disease burden, patients with comorbidities were less likely to have been prepared for transition than patients without comorbidities (65.4% vs. 79.2%) (**Tables 4**, **5**).

**Table 4:** Comparison of key transition care parameters between patients with and without comorbidities.

Parameter	With comorbidities (n = 26)	Without comorbidities (n = 24)	p-value
Prepared for transition, n (%)	17 (65.4)	19 (79.2)	–
Interval pediatric–adult visit, months	6.88 ± 9.69 (2.0)	2.90 ± 3.72 (2.0)	0.067
Transition rating (1–10)	6.96 ± 3.36 (8.0)	7.38 ± 2.10 (8.0)	0.783
Adult care rating (1–10)	8.69 ± 2.13 (9.5)	8.46 ± 1.74 (9.0)	0.331

1, strongly not recommended; 10, strongly recommended.

**Table 5:** Comparison of illness perception (B-IPQ) scores in patients with and without comorbidities.

B-IPQ domain (0–10)	With comorbidities mean ± SD (median)	Without comorbidities mean ± SD (median)	p-value
Impact of illness	6.60 ± 2.20 (7.0)	4.86 ± 2.80 (5.0)	**0.028**
Timeline	9.32 ± 1.70 (10.0)	9.24 ± 1.76 (10.0)	1.000
Personal control	6.20 ± 2.29 (6.0)	6.57 ± 3.08 (8.0)	0.385
Treatment control	6.60 ± 2.18 (6.0)	5.45 ± 3.05 (6.0)	0.345
Symptom severity	5.76 ± 2.93 (6.0)	3.24 ± 2.30 (2.0)	**0.004**
Concern	4.68 ± 2.32 (5.0)	3.71 ± 2.57 (4.0)	0.133
Illness coherence	5.83 ± 2.58 (6.0)	6.76 ± 2.39 (7.0)	0.211
Emotional representation	5.28 ± 2.44 (5.0)	3.95 ± 3.07 (4.0)	0.115

Bold values indicate statistical significance (p < 0.05).

### 3.5 Major problems and difficulties reported by patients and parents

Patients reported a wide range of difficulties during the transition from pediatric to adult care. Organizational and system-related problems most included inadequate coordination of the transfer process (reported by 7 patients, 14%). Three patients (6%) reported no opportunity to participate in choosing the adult care center, and one patient (2%) reported difficulties accessing medical documentation.

Psychosocial difficulties included a lack of psychological support during the transition period, reported by five patients (10%). Insufficient medical and administrative support during transition was also noted; seven patients (14%) reported limited involvement of healthcare professionals and three (6%) reported insufficient administrative support. In addition, five (10%) patients reported inadequate knowledge about their disease.

Only 17 patients (34%) reported direct communication between pediatric and adult centers during transition. As many as 19 (38%) did not know whether such contact had occurred or reported that it had not taken place.

The scope of medical documentation transferred was heterogeneous. While most patients received a summary of their medical history (n = 31, 62%), genetic test results, treatment plans, and vaccination records were transferred less frequently (≤14%) (**Table 6**).

**Table 6:** Scope of medical documentation transferred with the patient during transition to adult immunology care.

Type of documentation	n (%) (n = 50)
Complete medical record copy	31 (62.0)
Partial medical record copy	12 (24.0)
Summary of medical history	31 (62.0)
Imaging results	5 (10.0)
Vaccination record	3 (6.0)
Referral letter	3 (6.0)
Follow-up treatment plan	2 (4.0)
Genetic test results	7 (14.0)
Psychological assessment	1 (2.0)

Fourteen patients (28%) and three parents (17%) reported no difficulties related to the transition process.

### 3.6 Parents’ or caregivers’ perspective

Regarding transition preparation, 11 (61%) parents or caregivers reported that both they and their child had been prepared for transfer to adult care. Preparation of the parent or caregiver only was reported by 5.6% (n = 1), preparation of the child only by 11.1% (n = 2), and lack of preparation by 22.2% (n = 4).

Half of the parents or caregivers (n = 9) reported having the opportunity to choose the adult immunology center, while the remaining half did not. The most frequently reported factor influencing this choice was the recommendation from the pediatric center (n = 11, 61%). Six parents or caregivers (33%) indicated the multidisciplinary nature of the center, while five (27.7%) each cited distance, medical reputation, and recommendations from other patients.

Parents or caregivers also reported various difficulties during transition. Organizational challenges included lack of coordination (n = 5, 27.7%) and insufficient administrative support (n = 4, 22.2%). Psychosocial difficulties included lack of psychological support (n = 4, 22.2%). Difficulties related to the child’s preparation and engagement were also noted: four parents reported insufficient engagement of the child, one reported excessive involvement, and four reported insufficient disease-related knowledge. Three parents (16.6%) reported no difficulties.

### 3.7 Comparison of patient and parent or caregiver perspectives

A high level of agreement was observed between patients and parents regarding transition preparation and quality of adult care. However, parents rated the transition process itself significantly lower than patients (p = 0.02) (**Table 7**).

**Table 7:** Comparison of patient and parent or caregiver perspectives.

Aspect	Patients	Parents	p-value
Prepared for transition, n (%)	36 (72.0)	14 (77.8)	0.761
Transition rating (prepared), mean ± SD	7.89 ± 1.77	6.57 ± 3.14	**0.020**
Transition rating (unprepared), mean ± SD	5.29 ± 4.01	5.75 ± 3.50	0.061
Adult care rating, mean ± SD	8.83 ± 1.50	8.57 ± 1.74	0.626

1, strongly not recommended, the lowest value; 10, strongly recommended, the highest value.

Bold values indicate statistical significance (p < 0.05).

## 4 Discussion

This study represents the first analysis in Poland of experiences related to the transition from pediatric to adult care among young adults with inborn errors of immunity.

The most important finding of this study indicates that the way patients are prepared for transition has a significant influence on their emotional well-being. Unprepared patients had higher Emotional Representation scores (6.42 vs. 4.06; p = 0.019). This indicates greater emotional burden and more difficult adaptation to the adult care system.

Other B-IPQ domains, such as perceived illness consequences, disease understanding, or health-related concerns, did not differ significantly between prepared and unprepared patients. This suggests that transition preparation primarily affects emotional coping rather than disease knowledge, highlighting the need to incorporate emotional support and a sense of security as core components of transition care (15, 16).

This observation is consistent with qualitative studies conducted in Canada by Ouimet et al., who identified “entering adulthood” as a central element of the transition experience in patients with IEI (17). The authors emphasized that many patients have experienced long and emotionally burdensome diagnostic journeys, often associated with delayed diagnosis and feelings of being misunderstood. Such experiences may be reactivated during transition, particularly when the process is abrupt and inadequately supported (17). The present quantitative data provide empirical confirmation of these observations. The observed trend toward a greater sense of disease control among patients prepared for transition is consistent with the conceptual distinction between “transition” and “transfer” described by Tadros and Burns (6, 10). According to these authors, effective transition should involve the development of self-management skills, autonomy, and responsibility for one’s own health, whereas transfer alone represents merely an administrative change in the place of care (6, 10). In the context of the present study, patients who were not prepared for transition may have experienced the process as abrupt, unpredictable, and lacking adequate support. These findings highlight the importance of including psychologists or mental health professionals within transition care teams to address emotional readiness alongside medical preparation.

The median age at initiation of the transition process in the study group was 17 years. Compared with European data, this may indicate that transition preparation in Poland begins too late. In the RITA-ERN survey (9), transition preparation usually started at 16–18 years, while transfer to adult care occurred at 18–20 years. The consensus of the Italian Primary Immunodeficiency Network, French and British (Ready, Steady, Go) experiences emphasize the importance of initiating transition preparation as early as possible (15, 18, 19). The proposed models recommend starting discussions around the age of 14 and gradually preparing patients over several years, rather than concentrating efforts solely at the moment of reaching legal adulthood (15, 18, 19). Such an approach facilitates skill development and reduces stress associated with an abrupt transition to the adult healthcare system.

A particularly concerning finding of our study was the so-called comorbidity paradox. More than half of patients reported comorbid conditions, confirming the complex nature of IEI in adulthood. These patients experienced a greater symptom burden and a greater impact of disease on daily functioning, yet they were less frequently prepared for transition than patients without comorbidities.

This paradox may reflect several systemic factors. First, patients with comorbidities often require multidisciplinary care, which, as noted by Ouimet et al. (17), can result in “fragmented transitions” across multiple clinics, with no single team assuming overall responsibility for transition coordination. In Poland, immunology, rheumatology, pulmonology, and other specialties frequently operate in separate centers with limited inter-specialty communication.

This phenomenon may also stem from organizational challenges and the tendency of pediatric teams to prioritize acute clinical issues. However, the present findings suggest that such an approach may be counterproductive, as patients with the most complex disease profiles are likely to require the most comprehensive transition preparation.

Although most patients reported having received some form of transition preparation, analysis of preparation methods revealed substantial variability and significant gaps. The most common forms of preparation consisted of conversations with a physician or nurse. Only six patients reported a personal visit to the adult care center, and an equally small number reported the use of educational materials. This suggests that preparation was largely informational in nature and less focused on developing practical skills or familiarizing patients with the adult care environment. These observations are consistent with findings from Canadian studies indicating that the provision of information alone is insufficient (17). Effective transition also requires emotional support, confidence-building, and the development of skills necessary to navigate the healthcare system independently (15, 16). This may explain why, despite a relatively high proportion of patients reporting some form of preparation, nearly one-third still felt unprepared.

Fewer than half of the patients reported having the opportunity to choose their adult immunology center. Decisions were most often influenced by physician recommendations and geographic proximity, and less frequently by patient preferences. Limited autonomy in this area may negatively affect patients’ sense of control and contrasts with the concept of “true transition,” which emphasizes active patient participation in decision-making (15, 16, 20). Similar observations regarding limited autonomy during transition and its long-term consequences, including persistent dependence on parents after transfer to adult care, have been reported in qualitative studies involving patients with chronic conditions (17, 21).

The study also demonstrated a significant association between transition preparation and patient independence. Prepared patients were more likely to attend medical appointments without parental presence and demonstrated greater autonomy in managing their healthcare. Qualitative studies have similarly shown that, even after formal transfer, many patients continue to rely heavily on parents for treatment management and care organization (17, 20).

Comparison of patient and parent perspectives revealed high concordance regarding assessments of transition preparation, adult care quality, and patient independence. However, parents evaluated the transition process itself less positively than patients. This difference may reflect the emotional burden experienced by caregivers and their greater awareness of organizational barriers, as described in previous studies (17, 20). At the same time, both groups rated post-transition care highly, reinforcing the notion that difficulties primarily concern the transition phase rather than adult care itself.

Analysis of transferred medical documentation revealed a lack of standardization. Although most patients received a summary of their medical history, other key elements—such as genetic test results and follow-up treatment plans—were transferred much less frequently. In patients with IEI, the absence of such information may substantially hinder continuity of care and increase the risk of medical errors. Similar challenges have been reported in European and Asian studies, where lack of time and resources were identified as major barriers to comprehensive documentation transfer (9, 21). Models involving a dedicated transition coordinator, such as those proposed in Spain, may reduce this variability and improve continuity of care (7).

Data regarding communication between pediatric and adult care centers indicate significant deficiencies in coordination. A substantial proportion of patients reported either a lack of communication between centers or uncertainty as to whether such communication had occurred. This issue represented the most frequently reported difficulty among both patients and parents. These findings are consistent with international reports. Patients with IEI often require care from multiple specialists, and the absence of clearly defined responsibility for transition coordination may lead to fragmentation of care (16, 17).

One of the most consistently recognized components of effective transition models in rare and chronic diseases is the presence of a dedicated transition coordinator. Such models, recommended by ERN RITA and described in Italian and French consensus documents, assign responsibility for ensuring process continuity, facilitating inter-center communication, and supporting patients and families throughout transition (9, 15, 18). Implementation of this role within the Polish healthcare system may represent an important step toward improving the quality of transition care for patients with IEI.

Continuity of treatment during transition represents a major safety concern in IEI, particularly among patients receiving immunoglobulin replacement therapy or biological treatment (8). In our cohort, some patients experienced gaps in care lasting longer than 12 months, potentially increasing the risk of treatment interruption. According to the other authors (7, 20), the pediatric team should remain responsible for the patient until the first adult immunology appointment has been confirmed and care has been formally transferred. To minimize gaps in care, the first adult appointment should ideally be scheduled before the final pediatric visit. Joint consultations or meetings involving pediatric and adult care teams may further improve continuity of care.

### 4.1 Study limitations and future perspectives

This study has several limitations. The relatively small sample size (50 patients and 18 parents) limits statistical power. Although all participants had clinically significant disease, detailed diagnostic classifications were not collected in a format allowing diagnosis-specific analyses. In addition, the lack of objective health outcomes (e.g., hospitalization rates or complication indices) restricts assessment of the true clinical impact of transition preparation. The questionnaire did not assess the number, duration, structure, or quality of transition preparation visits. Consequently, it was not possible to determine whether “prepared” patients received a comprehensive transition program or only a brief discussion. Therefore, classification of patients as “prepared” or “unprepared” was based on self-report rather than objective criteria or standardized readiness assessment tools. Patients were recruited consecutively during outpatient visits. However, because participation was anonymous and voluntary, the response rate could not be determined. Patients less engaged in adult immunology care, including those lost to follow-up after transfer, were likely underrepresented in the study population.

Despite these limitations, this study provides the first Polish data on transition experiences among patients with IEI and establishes a foundation for future prospective, multicenter studies. The findings also highlight an urgent need to develop national guidelines for the transition of patients with IEI in Poland. Such guidelines should address the specific needs of patients with rare diseases, particularly those with comorbidities, emphasize early and gradual transition preparation, standardize medical documentation, define the role of a transition coordinator, and incorporate emotional support alongside the development of health-related competencies.

## 5 Conclusion

This study represents the first comprehensive analysis in Poland of the transition from pediatric to adult immunology care among young adults with inborn errors of immunity. The findings demonstrate that the transition process remains heterogeneous and frequently insufficiently structured.

A key finding was the significant association between lack of transition preparation and greater emotional burden among patients. Of particular concern was the observed comorbidity paradox, whereby patients with more complex clinical profiles—who require intensive, multidisciplinary care—were less likely to receive transition preparation.

The study also identified major gaps in coordination between pediatric and adult care centers, limited patient autonomy in selecting adult care providers, and a lack of standardization in the transfer of medical documentation. These findings provide a foundation for organizational strategies aimed at improving the quality and continuity of care for patients with inborn errors of immunity.

## Data Availability

The raw data supporting the conclusions of this article will be made available by the authors, without undue reservation.
