# Supplementary material for: Patient and caregiver perspectives on transition from pediatric to adult care in inborn errors of immunity: a Polish multicenter survey
Source: Front Immunol. 2026 Jun 26;17:1816943. doi: 10.3389/fimmu.2026.1816943 (PMC13349919; doi:10.3389/fimmu.2026.1816943)
Supplement: Supplementary file 1 [file Table1.docx]

**Transition Care – Patient Questionnaire**

Please answer the following questions:

1. Year of birth: ...............................
2. Education level:

- Primary
- Secondary
- Higher/University

1. Place of residence:

- Rural area
- City <50,000 inhabitants
- City 50–100,000 inhabitants
- City 100–500,000 inhabitants
- City >500,000 inhabitants

1. Sex:

- Female
- Male
- Other

1. What is the reason for your immunological care?

- Inborn error of immunity
- Autoinflammatory disease

1. Do you have any other chronic diseases?

- YES, please specify: ......................
- NO

1. Which type of pediatric center provided your care as a child? (multiple answers possible)

- Research institute
- Academic/university hospital
- District/regional hospital
- District/regional outpatient clinic
- General practitioner/family physician
- I do not know / I do not remember

1. Were you prepared at the pediatric center for transfer to an adult immunology center?

- YES
- NO

1. If yes (question 8), please describe what the preparation involved (multiple answers possible):

- Consultations with a physician
- Consultations with a nurse
- Consultations with a psychologist
- Consultation with another healthcare professional, please specify: .........................
- Provision of educational materials
- Referral/introduction to the adult immunology center
- Visit to the adult immunology center
- Other (please specify): ...................................

1. During medical appointments at the pediatric center, did you have the opportunity to consult with the physician without a parent/guardian present?

- YES
- NO

1. How old were you when discussions about transferring to a different center first took place during appointments?

.....................

1. At what age were you transferred to the adult immunology center?

.....................

1. Did you have the opportunity to choose your adult immunology center?

- YES
- NO

1. If given the opportunity to choose an adult center, what would be your primary criterion?

- Recommendation from the treating physician at the pediatric center
- Distance from place of residence
- Medical reputation
- Recommendation from other patients/acquaintances
- Availability of other specialists at the center
- Other ..........................................………

1. At what point did contact occur between the treating centers (pediatric and adult) during the patient transfer process?

- approximately 2 years before my transfer from pediatric to adult care
- approximately 1 year before my transfer from pediatric to adult care
- at the time of my transfer from pediatric to adult care
- after my transfer from pediatric to adult care
- the centers never contacted each other
- I do not know whether the centers were in contact

1. Which of the following medical documents did you have available at your first visit to the adult center? (multiple answers possible)

- Complete copy of medical records from the pediatric center
- Partial copy of medical records from the pediatric center
- Summary of medical history
- Electronic media with imaging results
- Genetic test results
- Psychological assessment report
- Vaccination record/summary
- Referral letter from the treating physician at the pediatric center
- Letter from a social worker
- Plan for ongoing medical care
- Other ..........................................….

1. What difficulties did you encounter during the transition from the pediatric to the adult immunology center? (multiple answers possible)

- Lack of understanding of one's own condition
- Lack of coordinated medical care during the transfer process
- Lack of support from the pediatric center medical staff during the transfer process
- Lack of support from the adult center medical staff during the transfer process
- Lack of support from administrative staff during the transfer process
- Lack of psychological support during the transfer process
- Lack of access to medical records
- Lack of opportunity to choose the place of ongoing care
- Excessive parental involvement
- Insufficient parental involvement
- Other .....................…………

1. How much time elapsed between the last visit at the pediatric center and the first visit at the adult center?

- <1 month
- 1–3 months
- 3–6 months
- 6–12 months
- 12–24 months
- >24 months

1. Did the transfer from pediatric to adult immunological care result in an interruption of chronic treatment (unplanned interruption of immunoglobulin substitution or biological therapy?)

- YES
- NO

1. During how many appointments at the adult center was a parent/guardian present?

- none
- only at the first appointment
- at several appointments
- at all appointments so far

1. On a scale of 1 to 10 (1 = very poor, 10 = excellent), how would you rate the transfer process from the pediatric to the adult immunology center?

1 2 3 4 5 6 7 8 9 10

1. On a scale of 1 to 10 (1 = very poor, 10 = excellent), how would you rate the care provided at the adult center?

1 2 3 4 5 6 7 8 9 10

1. On a scale of 1 to 10 (1 = would definitely not recommend, 10 = would definitely recommend) how likely are you to recommend immunological care at the pediatric center?

1 2 3 4 5 6 7 8 9 10

1. On a scale of 1 to 10 (1 = would definitely not recommend, 10 = would definitely recommend) how likely are you to recommend immunological care at the adult center?

1 2 3 4 5 6 7 8 9 10

1. Are you aware of, or have you heard about, structured transition programs from pediatric to adult care – the so-called “transition care”?

 YES

 NO

1. Please list the 3 most important aspects you would like to change in the transition process from pediatric to adult center care.

1....................................................................................………….

2....................................................................................………….

3....................................................................................………….

The Brief Illness Perception Questionnaire

For the following questions, please circle the number that best corresponds to your views:

1. **How much does your illness affect your life?**

0 1 2 3 4 5 6 7 8 9 10

no affect at all severely affects my life

1. **How long do you think your illness will continue?**

0 1 2 3 4 5 6 7 8 9 10

a very short time forever

1. **How much control do you feel you have over your illness?**

0 1 2 3 4 5 6 7 8 9 10

Absolutely no control extreme amountof control

1. **How much do you think your treatment can help your illness?**

0 1 2 3 4 5 6 7 8 9 10

not at all extremely helpful

1. **How much do you experience symptoms from your illness?**

0 1 2 3 4 5 6 7 8 9 10

no symptoms at all many severe symptoms

1. **How concerned are you about your illness?**

0 1 2 3 4 5 6 7 8 9 10

not at all concerned extremely concerned

1. **How well do you feel you understand your illness?**

0 1 2 3 4 5 6 7 8 9 10

don’t understand at all understand very clearly

1. **How much does your illness affect you emotionally? (e.g. does it make you angry, scared, upset or depressed?)**

0 1 2 3 4 5 6 7 8 9 10

not at all affected emotionally extremely affected emotionally

**Please list in rank-order the three most important factors that you believe caused your illness.**

**The most important causes for me:**

1. .................................................................

2. ...............................................................

3. ...............................................................

**Transition Care – Questionnaire for Parent/Guardian**

Please answer the following questions:

1. Year of birth.....................……….
2. Education level:

- Primary
- Secondary
- Higher/University

1. Place of residence:

- Rural area
- City <50,000 inhabitants
- City 50–100,000 inhabitants
- City 100–500,000 inhabitants
- City >500,000 inhabitants

1. Sex:

- Female
- Male
- Other

1. What is the reason for your child’s immunological care?

- Inborn error of immunity
- Autoinflammatory disease

1. Does your child have any other chronic diseases?

- YES, please specify: ......................
- NO

1. Which type of pediatric center provided care for your child? (multiple answers possible)

- Research institute
- Academic/university hospital
- District/regional hospital
- District/regional outpatient clinic
- General practitioner/family physician

1. Were you and/or your child prepared at the pediatric center for the transition to adult care?

- YES, both myself and my child
- YES, but only myself
- YES, but only my child
- NO

1. If yes (question 8), please describe what your (the parent/guardian’s) preparation involved (multiple answers possible):

- Consultations with a physician
- Consultations with a nurse
- Consultations with a psychologist
- Consultation with another healthcare professional, please specify: .........................
- Provision of educational materials
- Referral/introduction to the adult immunology center
- Visit to the adult immunology center
- Other (please specify): ...................................

1. During medical appointments at the pediatric center, did your child have the opportunity to consult with the physician without a parent/guardian present?

- YES
- NO

1. How old was your child when discussions about transferring to a different center first took place during appointments?

.....................

1. How old was your child at the first visit to the adult immunology center?

.....................

1. Did you have the opportunity to choose an adult immunology center for your child?

- YES
- NO

1. If given the opportunity to choose an adult center, what was your primary criterion?

- Recommendation from the treating physician at the pediatric center
- Distance from place of residence
- Medical reputation
- Recommendation from other patients/acquaintances
- Availability of other specialists at the center
- Other ..........................................………

1. At what point did contact occur between the treating physicians (the pediatrician and the adult care physician) during the patient transfer process?

- approximately 2 years before my transfer from pediatric to adult care
- approximately 1 year before my transfer from pediatric to adult care
- at the time of my transfer from pediatric to adult care
- after my transfer from pediatric to adult care
- the centers never contacted each other
- I do not know whether the centers were in contact

1. Which of the following medical documents did you have available for your child at the first visit to the adult center? (multiple answers possible)

- Complete copy of medical records from the pediatric center
- Partial copy of medical records from the pediatric center
- Summary of medical history
- Electronic media with imaging results
- Genetic test results
- Psychological assessment report
- Vaccination record/summary
- Referral letter from the treating physician at the pediatric center
- Letter from a social worker
- Plan for ongoing medical care
- I did not have any medical documentation
- Other ..........................................….

1. What difficulties did you encounter during the transition from the pediatric to the adult immunology center? (multiple answers possible)

- Lack of understanding of the condition
- Lack of coordinated medical care during the transfer process
- Lack of support from the pediatric center medical staff during the transfer process
- Lack of support from the adult center medical staff during the transfer process
- Lack of support from administrative staff during the transfer process
- Lack of psychological support during the transfer process
- Lack of access to medical records
- Lack of opportunity to choose the place of ongoing care
- Excessive involvement of the patient
- Insufficient involvement of the patient
- Other .....................…………

1. How much time elapsed between the last visit at the pediatric center and the first visit at the adult center?

- <1 month
- 1–3 months
- 3–6 months
- 6–12 months
- 12–24 months
- >24 months

1. Did the transfer of your child from pediatric to adult immunological care result in an interruption of chronic treatment (unplanned interruption of immunoglobulin substitution or biological therapy?)

- YES
- NO

1. On a scale of 1 to 10 (1 = very poor, 10 = excellent), how would you rate the transfer process of your child from the pediatric to the adult immunology center?

1 2 3 4 5 6 7 8 9 10

1. On a scale of 1 to 10 (1 = very poor, 10 = excellent), how would you rate the current care of your adult child at the adult center?

1 2 3 4 5 6 7 8 9 10

1. On a scale of 1 to 10 (1 = would definitely not recommend, 10 = would definitely recommend) how likely are you to recommend immunological care at the pediatric center?

1 2 3 4 5 6 7 8 9 10

1. On a scale of 1 to 10 (1 = would definitely not recommend, 10 = would definitely recommend) how likely are you to recommend immunological care at the adult center?

1 2 3 4 5 6 7 8 9 10

1. Are you aware of, or have you heard about, structured transition programs for patients from pediatric to adult care – the so-called “transition care”?

 YES

 NO

1. Please list the 3 most important aspects you would like to change in the transition process of a patient from pediatric to adult center care.

1....................................................................................………….

2....................................................................................………….

3....................................................................................…………
